# Development of a cost-effective quantitative in-house ELISA assay for screening anti-S1 IgG antibodies targeting SARS-CoV-2

**DOI:** 10.25122/jml-2023-0047

**Published:** 2023-06

**Authors:** Abdul-Sattar AL-Saeedi, Ahmed Sahib Abdulamir, Ghassaq Tariq Alubaidi

**Affiliations:** 1 Medical Microbiology Department, College of Medicine, Al-Nahrain University, Baghdad, Iraq; 2 Basic and Medical Sciences Branch, College of Nursing, University of Al-Ameed, Karbala, Iraq; 3 Medical Research Unit, College of Medicine, Al-Nahrain University, Baghdad, Iraq

**Keywords:** SARS-CoV-2, ELISA, S1 subunit

## Abstract

The RBD, S, and N proteins, the three main antigens of the SARS-CoV-2 virus, activate the host immune system and cause the formation of IgM and IgG antibodies. While IgM indicates an early, acute infection stage, IgG shows a past infection or persistent sickness. This study used an indirect ELISA assay that targets the S1 subunit of the SARS-CoV-2 S protein to create an in-house, qualitative serological test specific to COVID-19. A total of 60 serum samples were examined using ELISA for anti-SARS-CoV-2 IgG, and 50 of those results were positive. An additional 20 samples were taken from cases that occurred before the pandemic. For the in-house ELISA assay, a plasmid containing the gene coding for the S1 subunit was transformed into *E. coli* DH5ɑ bacterial cells and the protein was synthesized and purified. The purified protein was utilized to coat the ELISA plate, which was subsequently used to assess the levels of IgG among individuals with SARS-CoV-2 infection. The study found a significant association (p-value=0.01) between the in-house and the commercial anti-S1 subunit IgG antibodies kits. The in-house ELISA responded well, with a sensitivity and specificity of 75.0% and 88.89%, respectively. Furthermore, a library of SARS-CoV-2 recombinant S1 subunits was created by competent bacteria and may be employed for various tasks, such as creating diagnostic tools and scientific investigation. Overall, the in-house anti-SARS-CoV-2 human IgG-ELISA proved to be sensitive and specific for identifying IgG antibodies in patients exposed to SARS-CoV-2.

## INTRODUCTION

The RBD, S, and N proteins of SARS-CoV-2 are the primary antigens that cause a host immune response and the subsequent production of immunoglobulin A (IgA), immunoglobulin M (IgM), and immunoglobulin G antibodies (IgG) [1]. Mucosal immune responses to SARS-CoV-2 are reflected in the titer of secretory IgA. In contrast to IgG, indicative of a chronic illness or a prior infection, IgM suggests the early, acute infectious stage [1]. The temporal dynamics of these antibodies can vary across studies, even though IgM and IgG were more frequently detected than IgA in connection to SARS-CoV-2 antibodies. IgA and IgM were detected on day five (median), and IgG was detected on day fourteen (study) (median) [2].

Tests based on the S antigen have demonstrated cross-reactivity with the Middle East respiratory syndrome coronavirus (MERS-CoV). However, the S1 protein within the S antigen is significantly more specific than the S protein and exhibits 100% specificity for coronaviruses other than SARS-CoV [3]. This is because the S2 subunit has less antigenic variation and is more stable than the S1 subunit, which may explain the observed pattern. Tests based on the N protein showed cross-reactivity with SARS-CoV and MERS-CoV [4, 5].

Serological tests utilized for determining patient antibodies include rapid diagnostic tests (RDTs), enzyme-linked immunosorbent assays (ELISAs), chemiluminescent immunoassays (CLIAs), and neutralization assays. RDTs often take the form of lateral flow assays [6]. These tests are valuable for estimating the cumulative incidence of prior infections [7]. When combined with the patient's medical history, physical examination, and imaging results, the test outcomes can provide reliable information for diagnosing SARS-CoV-2 infection. However, these findings need a thorough analysis of asymptomatic individuals or regions with low disease prevalence. It is critical to be aware of the advantages, disadvantages, and utility of a particular test to be able to assess its diagnostic efficacy. Moreover, incorporating additional diagnostic tests that offer a detailed analysis of the results is essential, considering the implications for both the patient and society [8]. The study aimed to develop a COVID-19-specific qualitative serological test using an indirect ELISA assay targeting the S1 subunit of SARS-CoV-2 S antigens.

## MATERIALS AND METHODS

### Study design and samples collection

This study utilized a laboratory-based experimental design to develop a COVID-19-specific qualitative serological test using an indirect ELISA assay targeting the S1 subunit of SARS-CoV-2 S antigens. The study aimed to assess the accuracy, sensitivity, and specificity of the in-house designed ELISA assay compared to a commercial anti-S1 IgG antibody test. Twenty-six serum specimens were collected from individuals who tested positive for IgG antibodies, including ten samples obtained before the pandemic. Sample collection took place between January 4 and April 1, 2022.

### Plasmid transformation and ELISA measurement

The plasmid containing the gene code for the S1 subunit was transformed into *E. coli* DH5ɑ bacterial cells, followed by protein synthesis and purification. The purified protein was then used to coat the ELISA plate and then used for subsequent measurement of IgG concentrations in individuals with SARS-CoV-2 infection.

### Production of SARS-CoV-2 (2019-nCoV) spike S1 protein

#### Competent bacterial cell preparation

Competent *E. coli* cells were prepared by inoculating 10 ml of brain-heart infusion broth medium at 37 degrees Celsius for 24 hours. Four sterile petri plates were prepared and inoculated with 4 milliliters of the culture. The experiment was incubated overnight at 37 degrees Celsius. Sterile polypropylene tubes were used to inoculate 10 mL of LB broth with a single colony from the overnight culture. The tubes were placed on ice for 5-10 minutes to maintain the cells at a low temperature throughout the subsequent procedures. Afterward, the tubes were centrifuged at 4°C and 1600 g for 7 minutes. Each pellet was resuspended in 10 ml of the ice-cold solution of calcium chloride after the excess solution was removed. The cells were all meticulously resuspended and kept on ice.

#### Transformation of competent cells

Fifty µL of competent cells were added to 5 µL of vector in a 1.5 ml polypropylene tube. After gently mixing the contents of the tube, it was placed in an incubator on ice for 30 minutes. The cells were heat shocked by submerging the tubes in a 42°C water bath for 30 seconds after incubation. The tube was left on ice for an additional two minutes. The cells were cultured for 1 hour at 37 °C in 2 ml of the LB broth culture medium. The transformation culture was plated on Kanamycin LB medium and incubated at 37 °C overnight.

#### Protein expression cultures

The procedure was conducted over three days. On the first day, transformed-competent cells were added to the LB medium and cultured overnight at 37 °C. The following day, 10 mL polypropylene tubes were filled with 5 mL LB medium supplemented with kanamycin. The tubes were inoculated with a single colony using a disposable loop and incubated overnight at 37 °C. On the third day, the content of the tubes became cloudy due to bacterial growth and was transferred into a freshly autoclaved LB broth medium with kanamycin. The flask was covered with cotton and incubated at 37°C with regulatory shaking. The cell growth was monitored by measuring the optical density using a spectrophotometer (A_600_) until it reached 0.7–1, typically taking approximately 8 hours. Once A_600_ reached the desired level, 0.2 g/liter of IPTG (isopropyl β-D-1-thiogalactopyranoside) was added to activate the bacterial promoter that controls the transcription on the plasmid. This stimulation initiated the production of the target protein by the *E. coli* culture. The culture was then incubated overnight at 37 °C. To harvest the cells, they were centrifuged at 4000 x g for 20 minutes and frozen overnight at -20 °C.

#### Manual protein purification

The cleared *E. coli* lysates were prepared, and manual protein purification was conducted using a magnetic agarose-based protocol targeting the DYKDDDDK-tagged protein. The tubes were incubated for 5 minutes at room temperature with frequent vortexing to facilitate the elution of the purified protein.

After incubation, the tubes were centrifuged, and the supernatant containing the eluted protein was carefully collected and preserved. The magnetic agarose beads were removed and collected, leaving the purified protein in the supernatant behind. To neutralize the low pH of the eluate, 15 µL of neutralizing buffer was added for every 100 µL of the eluate.

### ELISA assay

#### Preparation of indirect ELISA

To prepare the indirect ELISA, the SARS-CoV-2 S1 protein was diluted to a final concentration of 2 µg/mL in 1X coating buffer, a sodium bicarbonate buffer solution with pH 9.6. The dilution was performed using the S Lab synthesis protein (Elabscience). The 96-well microtiter plates were coated with 100 µl of diluted S Lab synthesis protein and then incubated at four degrees Celsius for 12 hours. Following incubation, the plate received three washes in 250 µl of PBST (PBS with 0.1% Tween 20). The plate was then sealed with a plate sealer and incubated for 12 hours at 4 °C. The wells were washed three times in 250 µl of phosphate-buffered saline (PBS) washing solution. The coated wells received 200 µl of 1% bovine serum albumin (BSA) (blocking buffer), and the plate was sealed again with plate sealer, which was incubated overnight at 4 °C. Samples of serum were added in 100 µL after the plate was cleaned as previously mentioned.

#### In-house designed indirect ELISA kit optimization

ELISA plate was coated with five different concentrations of S1 subunit prepared by dilution with phosphate buffer saline (PBS) to determine the optimal detection concentration. A single serum sample obtained from a patient who tested positive for SARS-CoV-2 by RT-qPCR and exhibited a high IgG neutralizing antibody, as measured by the SUNLONG ELISA kit, was used for the optimization process. The IgG level in the serum sample was measured using the in-house designed indirect ELISA kit, and the absorbance was read at 450nm using BIOTEK ELISA Reader/USA.

### Statistical Analysis

Data analysis was performed using Microsoft Excel 2016, GraphPad Prism version 6, and SPSS software version 26. Descriptive statistics, such as mean and standard deviation, were calculated to estimate the IgG concentrations in the serum samples of patients. Pearson's correlation coefficient was employed to assess the statistical association or differences between the two groups, with significance determined at a p-value of <0.05. Graphs were generated using GraphPad Prism version 6 software.

## RESULTS

### Result of in-house designed indirect ELISA kit optimization

**Figure 1:**
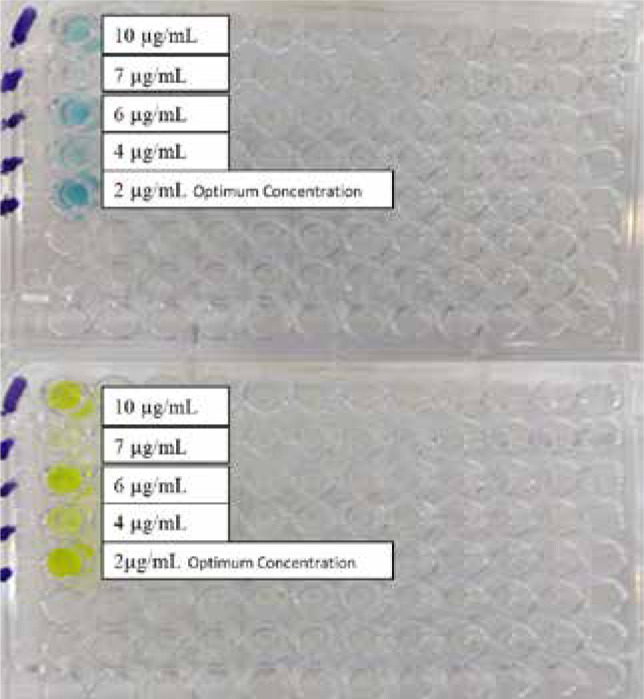
Five concentrations of SARS CoV-2

To optimize the in-house designed indirect ELISA kit, the ELISA plate was coated with five different concentrations of the S1 subunit. A single serum sample from a SARS-CoV-2 RT-qPCR positive patient with a high level of IgG neutralizing antibody, as measured by the SUNLONG ELISA kit, was used. The optimal concentration of the S1 subunit for detection was determined to be 2 µg/mL based on the highest optical density value obtained (OD = 2.71) at 450 nm, as shown in Figure 1.

### Limit of detection (LOD) and limit of quantification (LOQ)

The concentration ranges of neutralizing antibody levels in samples were determined using the in-house SARS-CoV-2 S1 subunit neutralizing ELISA test. The assay provided information on both the upper and lower limits of the neutralized antibody levels. The limit of detection (LOD) for the in-house designed ELISA assay was 23.20 ng/mL, and the limit of quantification (LOQ) was 70.31ng/mL. The commercial ELISA assay had a LOD of 67.29 ng/mL and a LOQ of 203.93 ng/mL. The samples included the top and lower limits of the quantities of neutralized antibodies as determined by the Human SARS-CoV-2 Spike Protein S1 IgG (S1-IgG) ELISA assay.

### Standard curve for Human SARS-CoV-2 Spike Protein S1 IgG (S1-IgG) ELISA assay

**Figure 2:**
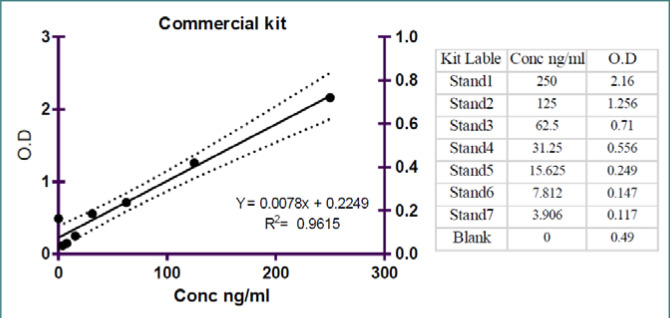
Standard curve of commercial ELISA kit

Figure 2 illustrates the standard curve generated using a commercial ELISA kit for the Human SARS-CoV-2 Spike Protein S1 IgG (S1-IgG) ELISA assay. The scatter plot presents the average OD absorbance measurements (x-axis) corresponding to different log concentration levels (y-axis) of SARS-CoV-2 neutralizing antibody standards. The data points on the graph form a straight line, representing the standard curve. Statistical analysis revealed a strong inverse relationship between the OD absorbance and the log level of neutralizing antibodies, as evidenced by a coefficient of determination (R2) value of 0.9615. The slope of the line was determined at 0.0078, and the intercept was 0.249. These results highlight the robust correlation between the OD absorbance and the concentration of neutralizing antibodies in the commercial ELISA assay.

### In-house ELISA assay standard curve

**Figure 3:**
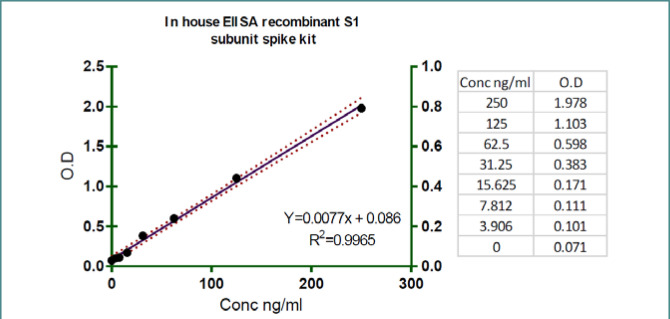
Standard Curve of in-house ELISA kit

Figure 3 displays the average OD absorption values for each SARS-COV-2 neutralizing antibody generated during the in-house neutralization ELISA experiment. The scatter plot displays the OD absorbance values (x-axis) and log concentration values (y-axis), with the standard curve depicted as a series of dots forming a straight line. The statistical analysis, using a coefficient of determination (R2), revealed a strong negative correlation between the OD absorption and the log concentration of neutralizing antibodies. The slope of the line was determined to be 0.0077, indicating the rate of change in OD absorption concerning the concentration. The coefficient of determination (R2) was 0.9965, indicating a high degree of linearity in the relationship. The intercept of 0.086 represents the estimated OD absorption value when the log concentration is zero.

### ELISA inter and intra assay

The precision or repeatability of the in-house designed ELISA assay was assessed by measuring the concentrations of positive samples in duplicate within each run (intra-assay) and across two different runs (inter-assay). The percent coefficient of variability (CV%) was calculated to determine the variability of the ELISA results, focusing on the concentration values in ng/mL. For the inter-assay variability, the CV% ranged from 2.79% to 7.79%, with an average CV% of 6.59%. The concentrations of the positive samples exhibited an average CV% that reflected both high and low values. The concentration means ranged from 96.345 ng/mL to 312.245 ng/mL. Regarding the intra-assay variability, the CV% ranged from 1.39% to 16.35%, with an average CV% of 7.18%. The concentration means showed a wider range of variation within the positive samples.

### In-house IgG concentration vs. commercial IgG concentration (Pearson’s correlation)

**Figure 4:**
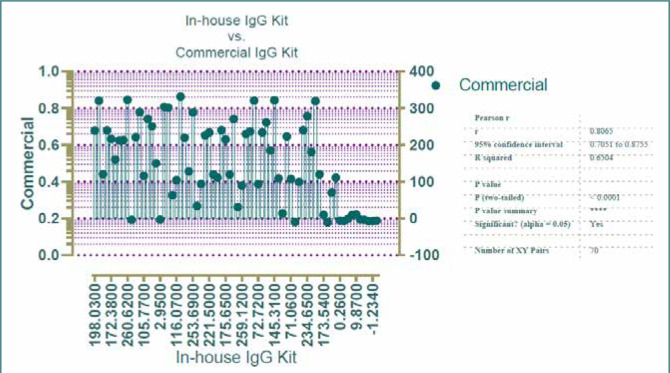
In-house IgG vs. commercial IgG concentrations

The relationship between the in-house anti-S1 subunit IgG neutralizing antibody kit and the commercial anti-S1 subunit IgG neutralizing antibody kit was evaluated using Pearson's correlation coefficient analysis. A total of 60 serum specimens were tested for IgG concentrations using both kits. The analysis revealed a significant association between the IgG concentrations measured by the two kits, with a correlation coefficient ranging from 0.088 to 0.578 at a 95% confidence interval. The p-value of 0.01 indicates a statistically significant correlation (Figure 4).

### Overall accuracy, sensitivity, and specificity of the in-house designed ELISA assay

The overall performance of the in-house designed ELISA assay was evaluated in terms of accuracy, sensitivity, specificity, positive predictive value (PPV), and negative predictive value (NPV). The assay demonstrated a sensitivity of 75.00% (95% CI 63.40% to 84.46%) and a specificity of 88.89% (95% CI 65.29% to 98.62%). The positive predictive value was 96.43% (95% CI 87.89% to 99.01%), while the negative predictive value was 47.06% (95% CI 67.79% to 85.87%). The overall accuracy of the test was 77.78% (95% CI 67.79% to 85.87%).

### Results of in-house designed indirect ELISA kit and Human SARS-CoV-2 Spike Protein S1 IgG (S1-IgG) ELISA kit

In addition, serum samples from all 60 subjects enrolled in the study were analyzed for anti-SARS CoV-2 IgG antibodies using both kits. Descriptive statistics were calculated for different severity groups. For mild serum samples, 18 out of 20 were positive for IgG when measured by the in-house designed kit, with a mean concentration of 200.57 ng/ml (SD 99.03), while 17 samples were positive with a mean concentration of 222.85 ng/ml (SD 75.51) when measured by the commercial kit. Similarly, all 20 moderate serum samples tested positive when measured by the in-house designed kit, with a mean concentration of 172.77 ng/ml (SD 81.03), and 18 samples were positive with a mean concentration of 182.30 ng/ml (SD 91.4) when measured by the commercial kit. Among the severe serum samples, 16 were positive with a mean concentration of 185.78 ng/ml (SD 82.9) using the in-house designed kit, while 17 samples were positive with a mean concentration of 156.8 ng/ml (SD 102.07) using the commercial kit. Finally, all 10 pre-pandemic serum samples tested negative when measured using the in-house designed and commercial kits.

## DISCUSSION

The S1 antigen is more specific than the S antigen for detecting coronaviruses other than SARS-CoV because the S antigen is cross-reactive with MERS-CoV [2]. The SARS-CoV-2 S1 protein ELISA protocol developed in this study provided an accurate screening method for anti-S1 IgG antibodies in individuals who have recently been infected with SARS-CoV-2 or have been previously exposed to or vaccinated against it. This protocol offers a cost-effective ELISA technique with qualitative and quantitative capabilities, with an estimated cost of no more than 3 USD per individual test.

Controlling the COVID-19 pandemic and preventing future ones require effective SARS-CoV-2 screenings. Direct detection techniques like RT-PCR and antigen tests are excellent at identifying viral nucleic acid (RNA) and surface proteins when the virus is actively reproducing, but their detection rates drastically decrease once the virus has stopped [9]. S-protein is employed in many vaccines because it is thought to be a key antigen of virus-neutralizing antibodies [10], so an in-house ELISA kit can be used as an effective tool for screening anti-SARS-CoV-2 IgG antibodies in vaccinated people.

In the current investigation, an in-house ELISA protocol for the SARS CoV-2-S1 protein was employed to evaluate the qualitative agreement of the results compared to a commercial anti-S1 IgG antibody test, which served as the gold standard assay.

The optimization of the in-house indirect ELISA kit involved coating the ELISA plate with different concentrations of the S1 subunit. The optimal concentration of the S1 subunit was determined to be 2 µg/mL based on the best result of optical density (OD = 2.71) at 450 nm. The molecular weight of the S1 subunit has an essential role in the concentration of the coating protein. The protein concentration range used in this study for coating the ELISA plate with the S1 subunit is consistent with the recommendations provided in The Immunoassay Handbook. The range of 1-10 µg/mL in a volume of 50-100 µL is considered suitable for saturating accessible sites on a polystyrene plate [11]. This finding aligns with a study by Daniel Stadlbauer *et al*. in 2020 in the USA, where a recombinant trimeric SARS-CoV-2 S protein was coated at a concentration of 2 µg/mL [12]. Another investigation used a concentration of 1 µg/mL for coating the S1 recombinant protein [13]. In a study by Brandi Freeman *et al*. in 2020 in Atlanta, a protein concentration of 0.15 µg/mL was used, which was shown to provide a saturating signal in recovering sera even at higher levels of serum dilution [14]. The variation in the protein concentrations used in different studies may be attributed to factors such as the molecular weight of the protein used and the specific requirements of the assay.

### ELISA inter and intra assay

The percent coefficient of variability (CV%) of the ELISA assay for the concentration in ng/ml ranged from 2.79 to 7.79% for inter-assay, with an average CV% of 6.59%. For intra-assay, the CV% ranged from 1.39 to 16.35%, with an average CV% of 7.18 %. The concentrations mean ranged from 96.345 to 312.245 ng/ml. Moreover, values below 10% for intra-assay and below 15% for inter-assay are suitable and indicate reliable results [15].

Interestingly, the pre-pandemic samples exhibited minimal reactivity, especially in the S1-IgG ELISA, indicating a slight potential for cross-reactivity with pre-existing immunoglobulins. This is because the S1 subunit, which is more resilient than other spike protein components and has a high affinity for SARS-COV-2, is responsible. These findings are consistent with the study conducted by Carolina de la *et al*. in Panama in 2021, where most COVID-19 patients exhibited strong antibody reactivity towards the receptor binding domain and the whole spike protein. In contrast, the pre-pandemic specimens did not indicate reactivity [16]. Similarly, Shi *et al*. in 2020 in China emphasized the specificity of S1 antigen-based detection compared to the S antigen, which demonstrated cross-reactivity with MERS-coronavirus [3]. According to certain studies, human samples have been observed to have antibody cross-reactivity in response to many coronaviruses [17, 18].

### In-house IgG vs. commercial IgG concentrations (Pearson’s correlation)

Regarding the relationship between the in-house and the commercial anti-S1 subunit IgG antibody, we found important variations in their capacities to detect SARS-CoV-2 antibodies. Pearson's correlation analysis of IgG concentrations showed a significant difference (p-value=0.01) between the two kits, with a 95% confidence interval ranging from 0.088 to 0.578. This difference may be attributed to variations in the concentrations of the coated proteins. It also appears that the in-house ELISA kit, utilizing a recombinant protein, demonstrated a detection capacity that was not comparable to that of the commercial ELISA kit. A similar observation was found in another study that compared the in-house ELISA kit with MyBioSource ELISA Kit, and the outcomes revealed a significant difference between the two kits [19]. Additionally, there was no statistically significant difference between the immunodiagnostic kit and the in-house developed ELISA kit in the same study (p=0.313) [19].

### The overall accuracy, sensitivity, and specificity of the in-house designed ELISA assay

Serum samples from all 60 subjects enrolled in the study were analyzed for anti-SARS-CoV-2 IgG antibodies using the ELISA protocol. When IgG levels were measured in 20 mild serum samples, 18 were positive using the in-house-designed kit, and 17 were positive using the commercial kit. For 20 moderate serum samples, all were positive for IgG when measured with the in-house designed kit, and 18 were positive when measured with the commercial kit; for 20 severe serum samples, 16 were positive when measured with the in-house designed kit, and 17 were positive when measured with the commercial kit. Finally, the pre-pandemic 10-serum samples revealed negative results when measured by the in-house-designed and commercial kits.

In the current work, the in-house developed ELISA performed well with a sensitivity of 75.00% and a specificity of 88.89%. It also demonstrated a 96.43% positive predictive value and a 47.06% negative predictive value. Finally, the accuracy of the test was 77.78%. Among the 50 serum samples taken from SARS-CoV-2 infected people and the 10 serum samples from pre-pandemic individuals, 16 were confirmed negative by an in-house-designed kit for anti-S1 SARS-CoV-2 IgG (88.89% specificity). Several false-positive anti-S1 Abs sera in the in-house developed ELISA assay might occur due to the low level of LOD (23 ng/mL) calculated for the in-house ELISA kit compared to the LOD calculated for the commercial ELISA kit, which is 67 ng/mL. The in-house ELISA kit demonstrated moderate sensitivity and high specificity, with a few false-negative and false-positive results, possibly due to pipetting errors, sample quality, and processing. Moreover, the purity of the fixed S1 recombinant protein may negatively affect the sensitivity and specificity of ELISA results.

Our research findings align with the observations made by Eberhardt *et al*. in 2021, where they reported the sensitivities of two anti-S1 tests from Euroimmun and Immun-diagnostic as 77.13% and 89.23%, respectively [20]. Researchers in Marburg, Germany, successfully identified IgG antibodies against the S1 component of the SARS-CoV-2 spike protein in the serum of COVID-19 patients using a new indirect ELISA. These results show the high specificity, sensitivity, and precision of the SARS-CoV-2 ELISA, making it ideal for epidemiological investigations and for assessing the immunogenicity of existing vaccine competition since the spike protein is used as a protein of interest in most vaccines against SARS-CoV-2[13]. The sensitivity of the IgM and IgG ELISA in the other research was 83 and 65%, respectively, whereas the sensitivity of the total Ab ELISA was 93.1% [21]. Roy *et al*. (2020) conducted a study in the USA and found that IgG antibody specificity and sensitivity varied during the days following symptom onset, namely between days 8 and 14 (specificity 99.57%) and days 14 and 28 (76.67%). Both specificity and sensitivity reached 100% more than 21 days after symptom onset. Antibodies that target different regions of the S antigen or induce new antibody effector activities can potentially impact immunization outcomes [22]. In this study, using different fixed target S1 subunits in the in-house and commercial kits may have contributed to false-negative and false-positive results. The choice of the target region within the S antigen plays a critical role in determining the specificity and sensitivity of serological assays.

## CONCLUSION

Developing a library of SARS-CoV-2 recombinant S1 subunits has provided valuable resources for various applications, including diagnostic tools and research. The in-house anti-SARS-CoV-2 human IgG-ELISA demonstrated high sensitivity and specificity in identifying IgG antibodies in patients exposed to SARS-CoV-2. This assay holds promise for the development of more accurate and reliable serological tests, as well as for evaluating the immunogenicity of current vaccine candidates.

## Personal thanks

We would like to express our sincere gratitude and appreciation to the Central Public Health Laboratory team, particularly to Iman M. Aufi, the Director of the Microbiology Virology Department, for their invaluable cooperation and kindness throughout this study.
